# Mucin 1 (MUC1) is a novel partner for MAL2 in breast carcinoma cells

**DOI:** 10.1186/1471-2121-10-7

**Published:** 2009-01-28

**Authors:** Susan Fanayan, Mona Shehata, Annelies P Agterof, Michael A McGuckin, Miguel A Alonso, Jennifer A Byrne

**Affiliations:** 1 Molecular Oncology Laboratory, Oncology Research Unit, The Children's Hospital at Westmead, Locked Bag 4001, Westmead, 2145 NSW, Australia; 2 The University of Sydney Discipline of Paediatrics and Child Health, The Children's Hospital at Westmead, Locked Bag 4001, Westmead 2145, NSW, Australia; 3 Department of Pharmaceutical Sciences, Utrecht University, Utrecht, the Netherlands; 4 Epithelial Cancer and Mucosal Biology Laboratory, Mater Medical Research Institute, Mater Health Services, South Brisbane 4101 Qld, Australia; 5 Centro de Biología Molecular "Severo Ochoa", Universidad Autónoma de Madrid and Consejo Superior de Investigaciones Científicas, Cantoblanco, 28049-Madrid, Spain

## Abstract

**Background:**

The *MAL2 *gene, encoding a four-transmembrane protein of the MAL family, is amplified and overexpressed in breast and other cancers, yet the significance of this is unknown. MAL-like proteins have trafficking functions, but their molecular roles are largely obscure, partly due to a lack of known binding partners.

**Methods:**

Yeast two-hybrid screening of a breast carcinoma cDNA expression library was performed using a full-length MAL2 bait, and subsequent deletion mapping experiments were performed. MAL2 interactions were confirmed by co-immunoprecipitation analyses and confocal microscopy was employed to compare protein sub-cellular distributions. Sucrose density gradient centrifugation of membranes extracted in cold Triton X-100 was employed to compare protein distributions between Triton X-100-soluble and -insoluble fractions.

**Results:**

The tumor-associated protein mucin 1 (MUC1) was identified as a potential MAL2 partner, with MAL2/MUC1 interactions being confirmed in myc-tagged MAL2-expressing MCF-10A cells using co-immunoprecipitation assays. Deletion mapping experiments demonstrated a requirement for the first MAL2 transmembrane domain for MUC1 binding, whereas the MAL2 N-terminal domain was required to bind D52-like proteins. Confocal microscopy identified cytoplasmic co-localisation of MUC1 and MAL2 in breast cell lines, and centrifugation of cell lysates to equilibrium in sucrose density gradients demonstrated that MAL2 and MUC1 proteins were co-distributed between Triton X-100-soluble and -insoluble fractions. However co-immunoprecipitation analyses detected MAL2/MUC1 interactions in Triton X-100-soluble fractions only. Myc-MAL2 expression in MCF-10A cells was associated with both increased MUC1 detection within Triton X-100-soluble and -insoluble fractions, and increased MUC1 detection at the cell surface.

**Conclusion:**

These results identify MUC1 as a novel MAL2 partner, and suggest a role for MAL2 in regulating MUC1 expression and/or localisation.

## Background

Human MAL2, a 19 kDa protein with four transmembrane (TM) domains [1,2] is a member of the MAL protein family. The founding member MAL [3] resides in lipid rafts [4,5] and is required in apical vesicle transport [6-9]. The MAL family also includes less characterised members, including BENE, which is also a raft-associated integral membrane protein [10], plasmolipin, a 20 kDa proteolipid expressed in compact myelin and epithelial cells [11] and chemokine-like factor superfamily 8 (CKLFSF8), a novel regulator of EGF-induced signalling [12]. MAL2 was identified as a partner for tumor protein D52-like proteins through yeast two-hybrid (Y2H) expression screening of a human breast carcinoma library [1]. The MAL2 protein is now known to be expressed in many epithelial cell types, as well as peripheral neurons, mast cells and dendritic cells [13]. In HepG2 hepatoma cells, MAL2 resides exclusively within lipid rafts, and represents an essential component for indirect basolateral-to-apical transcytosis [2], where it shows a highly dynamic subcellular localisation [14]. MAL2 has also been reported to be distributed in both lipid raft and non-raft fractions in primary thyrocytes [15] and PC-3 prostate carcinoma cells [16], predicting additional, uncharacterised cellular functions for MAL2 outside lipid rafts.

The initial identification of *MAL2 *suggested its overexpression in breast cancer [1], which is supported by the *MAL2 *gene being found at chromosome 8q24, which is frequently gained in breast and other cancers [17]. Several studies have now identified *MAL2 *amplification and/or overexpression in breast cancer [18-22]. Overexpression of *MAL2 *has also been reported in other cancers, including primary ovarian carcinoma [23,24] and ascites [25], and pancreatic carcinoma [26], where *MAL2 *has since been employed as a discriminator of pancreatic carcinoma versus chronic pancreatitis [27]. Expression profiling has also indicated *MAL2 *overexpression in malignant pleural mesothelioma of the epithelial type [28], and in head and neck squamous cell carcinoma [29]. Immunohistochemical analyses first revealed differential MAL2 expression in renal carcinomas [15], with this being recently confirmed in chromophobe renal cell carcinoma versus oncocytoma [30].

Despite numerous reports of MAL2 overexpression in breast cancer, little is known about how increased MAL2 expression may provide an advantage to cancer cells. While MAL2 cellular localisation and function have been explored in previous studies [2,14,15], the only known MAL2 partners are members of the D52-like protein family [1]. Interactions between MAL2 and both D52 and D53 have since been identified in a large scale Y2H analysis [31], which supports further use of the Y2H system to analyse MAL2 function. We therefore carried out a Y2H screening of a breast carcinoma cDNA expression library [1,32] to identify novel MAL2 binding partners. One protein thus identified was mucin 1 (MUC1), a transmembrane protein expressed on the apical surface of epithelial cells [33] and overexpressed in multiple cancers [34], in part through *MUC1 *gene amplification [35]. Like other mucins, MUC1 protects and lubricates normal glandular epithelia, whereas MUC1 overexpression in cancer alters many cellular properties, including intercellular adhesion and immune recognition [33,34]. As MUC1 therefore represented a candidate MAL2 partner of particular interest, subsequent experiments were performed to confirm MAL2/MUC1 interactions, and examine their significance in breast epithelial and cancer cells. As we will describe, this work identifies a MAL2 as a cytoplasmic MUC1 partner which binds MUC1 in non-lipid raft fractions, and may regulate MUC1 expression and/or subcellular distribution.

## Methods

### Plasmid constructs

Bait proteins for use in the Y2H system were expressed from the pAS2-1 vector (Clontech, Mountain View, California, USA), and prey proteins were expressed from the pACT2 (Clontech) or pAD-GAL4 (Stratagene, La Jolla, CA, USA) vectors. The pAS2-1MAL2 bait construct was obtained by subcloning an *EcoRI-XhoI *fragment, representing the entire *MAL2 *cDNA insert, into the *EcoRI *and *SalI *sites of pAS2-1 vector. For the pAS2-1MAL and pACT2MAL constructs, a *MAL *cDNA insert was amplified by PCR amplification to introduce a 5' *NcoI *site, and a 3' *XhoI *site downstream of the stop codon, which was then subcloned into the corresponding or compatible sites in pAS2-1 and pACT2. Constructs including truncated versions of the *MAL2 *coding region were amplified by the polymerase chain reaction (PCR) using specific primers (Table 1). For the pAS2-1MAL2ΔN and pACT2 MAL2ΔN constructs, primers introduced a 5' *NcoI *site, and a 3' *XhoI *site downstream of the stop codon. For the pAS2-1MAL2ΔNTM1, pAS2-1MAL2ΔNTM12, pAS2-1MAL2ΔNTM123 and pAS2-1MAL2ΔNTM1234 constructs, primers introduced a 5' *EcoRI *site, and a 3' *XhoI *site downstream of the stop codon. All subcloned constructs were verified by DNA sequencing, with PCR-generated inserts being fully sequenced on one DNA strand. The pCR3.1/Myc-MAL2 expression construct has been previously described [2]. All constructs encoding D52-like fusion proteins, and the human breast carcinoma cDNA library constructed in the HybriZAP vector (Stratagene) have been previously described [1,32].

**Table 1:** Deleted MAL2 yeast two-hybrid constructs, primer sequences and MAL2 regions deleted

**Construct**	**PCR Primer Sequences (5'-3')**	**MAL2 region deleted**
pAS2-1MAL2ΔN pACT2MAL2ΔN	CATGCCATGGCCTACTCGGGCGCCTTCGTC CCGCTCGAGTTACGGTCGCCATCTTCGTAA	amino acids 1–34

pAS2-1MAL2 ΔNTM1	CCGGAATTCTCCTCCAATGTTCCTCTACC CCGCTCGAGTTACGGTCGCCATCTTCGTAA	amino acids 1–55

pAS2-1MAL2 ΔNTM12	CCGGAATTCCTCTCTGGAATGGTGGCT CCGCTCGAGTTACGGTCGCCATCTTCGTAA	amino acids 1–86

pAS2-1MAL2 ΔNTM123	CCGGAATTCGAAGCAGCAGCCACATCC CCGCTCGAGTTACGGTCGCCATCTTCGTAA	amino acids 1–119

pAS2-1MAL2 ΔNTM1234	CCGGAATTCGCTTTACGAAGATGGCGACCG CCGCTCGAGTTACGGTCGCCATCTTCGTAA	amino acids 1–169

### Yeast two-hybrid system and screening

Yeast cultures of the *Saccharomyces cerevisiae *Hf7c strain were grown at 30°C in standard liquid or solid media, based upon either rich YPD media (2% bacto-peptone, 1% yeast extract, 2% dextrose), or minimal SD medium (0.67% yeast nitrogen base without amino acids, 2% dextrose, with appropriate amino acid supplements) for expression library screening and direct interaction testing. For cDNA library expression screening, bait (pAS2-1MAL2) and human breast carcinoma pAD-GAL4 library plasmids were transfected simultaneously into Hf7c cells. Subsequent screening and the recovery of plasmid DNA from yeast cells were carried out as described [32]. For the direct testing of interactions, paired baits (pAS2-1 constructs) and preys (pACT2 or pAD-GAL4 constructs) were transfected into Hf7c cells as described [32]. Interactions between baits and preys were assessed by qualitatively determining HIS3 reporter gene activity [1].

### Antibodies

The BC2 (an anti-MUC1 VNTR epitope mouse IgG1 monoclonal) and FITC-BC2 antibodies have been previously described [36]. Rabbit polyclonal c-Myc (A-14) and CAV1 antibodies were purchased from Santa Cruz Biotechnology (Santa Cruz, CA, USA) and BD Biosciences (BD Biosciences, San Jose, CA, USA), respectively. Affinity-purified D52 rabbit polyclonal antibody has been described previously [37]. Peroxidase-conjugated donkey anti-rabbit and anti-mouse, FITC-conjugated donkey anti-mouse and CY3-conjugated donkey anti-rabbit secondary antibodies were purchased from Jackson ImmunoResearch, Inc (West Grove, PA, USA).

### Preparation and affinity purification of polyclonal MAL2 antisera

For the production of sheep antisera, one sheep was injected subcutaneously with 2 mg coupled MAL2 C^165^-P^176 ^peptide antigen on 2 occasions, spaced by 3 weeks. For the production of rabbit antisera, two rabbits were injected subcutaneously with 0.5 mg coupled N^13^-V^24 ^and C^165^-P^176 ^MAL2 peptide antigens on 2 occasions, spaced by 2 weeks. Antisera were affinity-purified using relevant MAL2 peptides as previously described [37].

### Human breast cell lines

The MDA-MB-435 (a kind gift from Dr Janet Price, MD Anderson Cancer Centre, Houston, TX), MDA-MB-453, SK-BR-3 and MCF-7 breast cancer cells were cultured as described in the American Type Culture Collection Catalogue. MCF-10A cells are described in the American Type Culture Collection Catalogue and were cultured as described [38].

### Derivation of stably-transfected cell lines

The Myc-tagged MAL2 expression construct was stably transfected into MCF-10A cells. Cells were seeded at approximately 60% confluence in 100 mm dishes and transfected 18 h later with 20 μg plasmid DNA using LipofectAMINE 2000 Reagent (Life Technologies, Inc., Gaithersburg, MD, USA), according to the manufacturer's instructions. After 24 h, G418 was added to a concentration of 1 mg/ml. Media were replenished every 2–3 days and a G418-resistant mixed population was selected 14 days post-transfection. MDA-MB-435 and MDA-MB-453 breast cancer cells were transfected by electroporation with a *MUC1 *cDNA containing 22 VNTR repeats in the pcDNA3 vector (Invitrogen) or the vector alone. Stable G418 resistant clones were isolated and MUC1 expression determined by flow cytometry.

### Preparation of total protein extracts and Western blot analyses

Cells were harvested in cold PBS, pelleted and washed twice in cold PBS. Total cell protein extracts were prepared by resuspending the pellet in SDS extraction buffer (125 mM Tris-HCl pH 6.8, 3% SDS, 5% 2-mercaptoethanol, 1 mM PMSF, and protease inhibitors [Roche, Basel, Switzerland]), which were then briefly sonicated. Samples were resolved using SDS-PAGE on 12.5% polyacrylamide gels, and electrotransferred to nitrocellulose filters (Millipore, Billerica, MA, USA). Protein loading was analysed using Ponceau S staining, and filters were blocked overnight at 4°C in 5% skim milk powder in TBS. Membranes were washed twice with TBS and incubated with either affinity-purified rabbit polyclonal MAL2 antisera (1/100), affinity-purified rabbit polyclonal D52 antisera (1/100), BC2 (1/100) or CAV1 antibody (1/2000) in 0.1% Tween 20 in TBS, for 2 h. Membranes were washed 3 times in 0.1% Tween 20 in TBS, and then incubated with horseradish peroxidase-conjugated donkey anti-rabbit or donkey anti-mouse secondary antibody (Jackson ImmunoResearch, Inc) (1/5000) for 2 h. Blots were washed 4 times with 0.1% Tween 20 in TBS, followed by 2 washes in TBS and antigen-antibody complexes were visualised by Western lightning chemiluminescent reagent (Perkin Elmer, Waltham, Massachusetts, USA).

### Co-immunoprecipitation analyses

MCF-10A cells were grown to 80% confluence in 100 mm dishes and washed with cold PBS. For each co-immunoprecipitation, proteins were extracted by scraping cells from 6 dishes into 0.15 ml lysis buffer per dish. For co-immunoprecipitation of MAL2 and D52 proteins, 10 mM Tris with 1 mg/ml Saponin (Sigma-Aldrich, St. Louis, MO, USA) was employed as a lysis buffer [39]. For co-immunoprecipitation of MAL2 and MUC1 proteins, SDS lysis buffer (125 mM Tris-HCl pH 6.8, 3% SDS, 5% 2-mercaptoethanol, 1 mM PMSF, and protease inhibitors (Roche) was employed. Protein A-Sepharose beads (Sigma-Aldrich) plus rabbit MAL2 antisera (1/50), either alone or with 1 μg/ml synthetic peptides N^13^-V^24 ^and C^165^-P^176^, or protein G-agarose beads (Sigma-Aldrich) plus BC2 (1/50) were added to cell lysates and incubated on a rotary mixer at 4°C for 16 h. Beads were washed 4 times with 1 ml lysis buffer, followed by a final wash with PBS. Eluted proteins (15 μl) were separated using SDS-PAGE on 12.5% polyacrylamide gels and electrotransferred to nitrocellulose filters (Millipore) for Western blot analyses, as above.

### Immunofluorescent labelling of breast cell lines

Cell lines were cultured to near confluence, and harvested by trypsinisation. Cells were diluted 3- to 10-fold and cultured overnight on glass coverslips. For immunofluorescent staining, cells were washed twice with PBS, and fixed in 4% paraformaldehyde supplemented with 0.1% saponin for 20 min at RT. Cells were washed twice with PBS, and incubated at RT for 2 h with affinity-purified rabbit MAL2 (1/50), D52 (1/100) antisera or mouse BC2-FITC (40 μg/ml), in 0.1% bovine serum albumin (BSA) in PBS. Primary antibody was omitted in control incubations. Cells were washed twice with PBS and incubated with a CY3-conjugated donkey anti-rabbit secondary antibody (1/500) (Jackson ImmunoResearch) in 0.1% BSA in PBS, for 1 h in the dark. Cells were washed again and DNA was counterstained with 10 nM DAPI (Sigma-Aldrich). Following 2 washes in PBS, cells were mounted in DAPCO (Sigma-Aldrich) prepared according to the manufacturer's instructions. Images were taken using a TCS SP2 Laser Scanning Confocal microscope (Leica Technologies, Wetzlar, Germany), using a 63× oil-immersion objective and a 4× zoom factor.

### Membrane fractionation analyses

Triton X-100 soluble and insoluble fractions were prepared essentially as described [40]. Cells grown in 4 × 100 mm dishes were treated with 20 mM methyl β-cyclodextrin (MβCD) at 37°C for 30 min. Cells were then rinsed with PBS and lysed for 20 min in 1 ml 25 mM Tris-HCl pH 7.5, 150 mM NaCl, 5 mM EDTA, 1% Triton X-100 at 4°C. The lysate was brought to 40% sucrose in a final volume of 4 ml, placed at the bottom of a 12 ml tube, and then layered with 6 ml 30% sucrose followed by 2 ml 5% sucrose, made in the same buffer without Triton X-100. Gradients were centrifuged for 22 h at 36,000 rpm at 4°C in a Beckman SW41 rotor. Fractions of 1 ml were harvested from the top of the tube and aliquots were subjected to Western blot analyses.

## Results

### Yeast two-hybrid screening identifies MUC1 as a putative MAL2 partner

**Figure 1:**
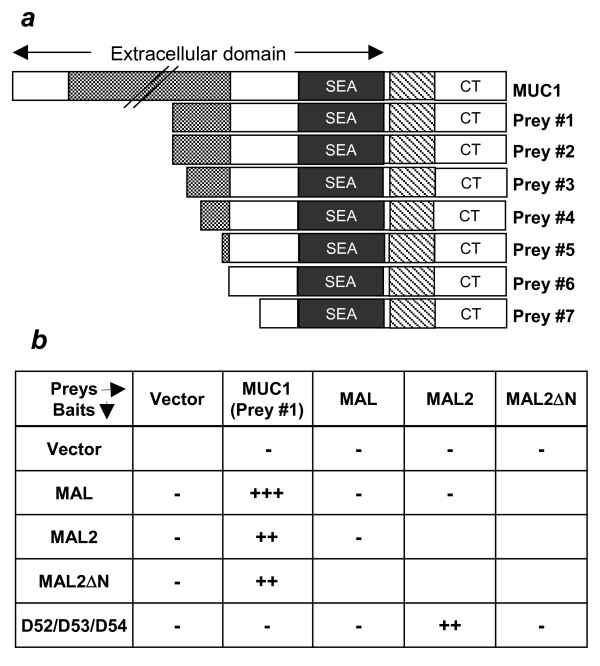
**(a) Schematic diagram of MUC1 preys (prey#1-prey#7) isolated during Y2H screen. All prey proteins were predicted to include the SEA module, the transmembrane domain (TM) and the cytoplasmic tail (CT)**. The length of the extracellular tandem repeat is not shown to scale in full-length MUC1. **(b) **Deleting the MAL2 N-terminus does not affect its binding to MUC1 but abrogates its interaction with D52-like proteins. Interactions were tested between MAL, MAL2 or MAL2ΔN, and MUC1 (prey#1) or D52-like proteins in Hf7c cells using the Y2H system. (-) indicates no detectable growth of co-transformants on triple drop-out plates after 6–8 days at 30°C, (++) indicates growth after 3–4 days and (+++) indicates growth after 1–2 days. Identical results were obtained for all 3 D52-like baits, which were grouped together in a single column. Results shown are representative of those obtained in 3 independent experiments.

To identify MAL2 partners expressed in breast cancer tissue, a Y2H screen of a breast carcinoma cDNA expression library [1,32] was performed with a full-length MAL2 bait. Screening 5,000,000 cfu in Hf7c cells identified 72 Hf7c colonies that remained His+ on restreaking on solid SD/-Leu-Trp-His media. Sequencing the corresponding cDNA inserts identified 6 novel candidate MAL2 binding partners, of which MUC1 was most frequently isolated. DNA sequencing indicated that all 7 MUC1 prey constructs [preys #1–#7] encoded N-terminally truncated MUC1 proteins, which commonly included the SEA module, the TM domain and the cytoplasmic tail (Figure 1a). Testing whether MUC1 (prey#1, Figure 1a) could reproducibly bind MAL2 and/or the related protein MAL showed that MUC1 could bind both MAL2 and MAL baits in the Y2H system (Figure 1b). In contrast, D52-like baits bound MAL2 prey as previously reported [1], but not MAL (Figure 1b). Since MAL and MAL2 sequences differ in their N-terminal regions [1] and a full-length MAL2 prey was originally isolated using a D54 bait [1], the MAL2 N-terminal domain (M^1^-T^34^) was hypothesised to represent the interaction interface with D52-like proteins. Deleting this region abolished interactions with all D52-like proteins tested, but did not affect interactions with MUC1 (Figure 1b), suggesting that MUC1 and D52-like proteins bind discrete regions of MAL2.

### Derivation of polyclonal antisera which specifically recognise MAL2 protein

**Figure 2:**
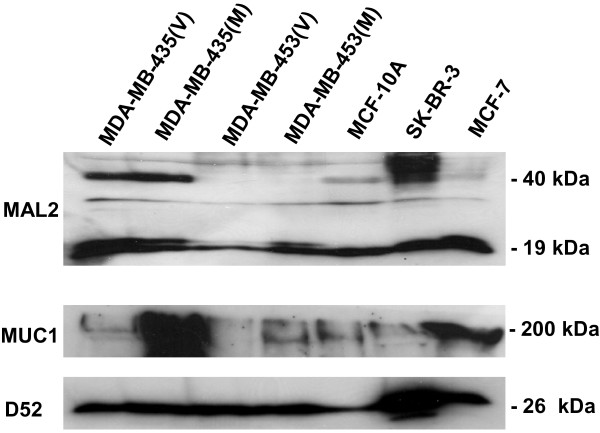
**MAL2 antisera detect glycosylated and non-glycosylated MAL2 forms in human breast cell lines, as indicated above the top panel.** Total protein extracts were subjected to Western blot analysis using rabbit MAL2, D52 and MUC1 antisera, as indicated to the left of each panel. Sizes of detected proteins are indicated at the right. Results shown are representative of at least 3 independent experiments.

To confirm MAL2/MUC1 interactions in other systems, MAL2 antisera were generated in two species. Polyclonal antisera were generated in sheep and targeted the MAL2 C-terminus (C^165^-P^176^). An N-terminal MAL2 peptide (N^13^-V^24^) [2] as well as the C-terminus (C^165^-P^176^) were also targeted for polyclonal antibody production in rabbit [1]. Both peptide sequences are poorly conserved in other MAL-like proteins [1]. Rabbit MAL2 antisera were employed in Western blot analyses of total protein extracts from a panel of human breast carcinoma cell lines, as well as MCF-10A breast epithelial cells. A MAL2 species of the predicted molecular weight of 19 kDa was detected in extracts from MDA-MB-435 and MDA-MB-453 cell lines stably transfected with a MUC1 expression vector (M) or the corresponding vector (V), and from MCF-10A, SK-BR-3 and MCF-7 cells. This is in agreement with previous detection of the 19 kDa MAL2 species in other cell types [2,13]. Higher molecular weight MAL2 species were also detected (Figure 2), and are likely to represent glycosylated MAL2 forms [1,2,15].

### Co-immunoprecipitation of MUC1 and MAL2 from MCF-10A/Myc-MAL2 cells

**Figure 3:**
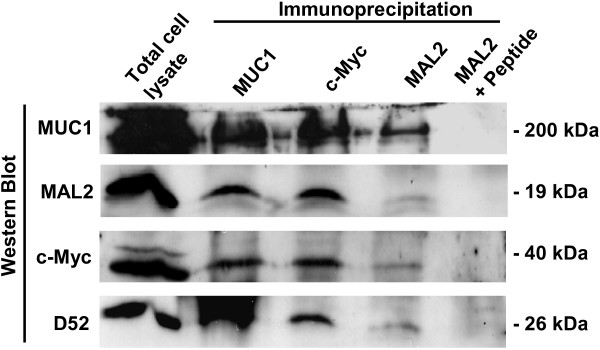
**Co-immunoprecipitation analyses verifying interactions between MAL2 and MUC1**. Total cell lysate from MCF-10A/Myc-MAL2 cells was immunoprecipitated with MUC1, c-Myc, or rabbit MAL2 antisera, either alone or with MAL2 peptides, as indicated above the top panel. Total cell lysate and immunoprecipitates were separated by SDS-PAGE and subjected to Western blot analyses with antisera against MUC1, MAL2, c-Myc and D52, as indicated at the left. Sizes of detected proteins are indicated at the right. Results shown are representative of at least 3 independent experiments.

Co-immunoprecipitation analyses were used to confirm interactions between MAL2 and MUC1 proteins in MCF-10A breast epithelial cells, stably-transfected with a Myc-tagged MAL2 construct (MCF-10A/Myc-MAL2 cells). Protein extracts were immunoprecipitated with either MUC1, c-Myc or MAL2 antisera, the latter in the absence or presence of MAL2 peptides. Immunoprecipitated proteins were then subjected to Western blot analyses with MUC1, MAL2, c-Myc and D52 antisera. These analyses demonstrated that MUC1 monoclonal antibody immunoprecipitated MUC1 and co-immunoprecipitated MAL2, as demonstrated by both MAL2 and c-Myc antisera, and that c-Myc and MAL2 antisera immunoprecipitated MAL2 and co-immunoprecipitated MUC1 (Figure 3). Interactions between MUC1 and MAL2 proteins were also reproducibly demonstrated in MCF-7 cells and MUC1-transfected MDA-MB-435 cells (data not shown). As expected, D52 co-immunoprecipitated with MAL2 and c-Myc antisera, and interestingly with MUC1 as well (Figure 3), suggesting that MUC1 and D52 may form a multi-molecular complex with MAL2. Inclusion of MAL2 peptide abolished immunoprecipitation of MAL2 and co-immunoprecipitation of both MUC1 and D52 (Figure 3).

### Mapping the MAL2 region required for MUC1 binding

**Figure 4:**
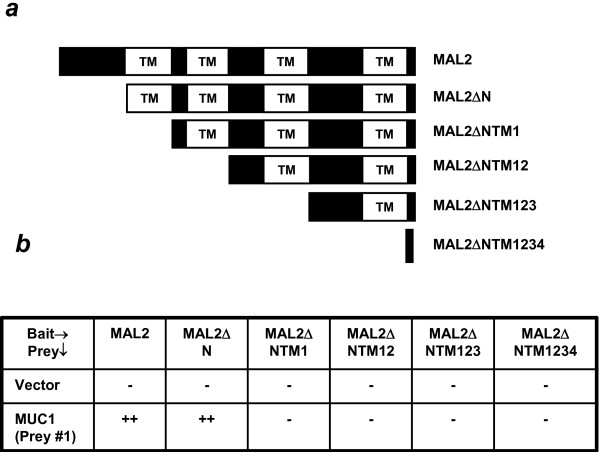
**(a) Schematic representation of N-terminally-deleted MAL2 proteins (defined in Table **1**) employed in Y2H analyses**. **(b) **Results of testing interactions between full-length and deleted MAL2 bait proteins and MUC1 (prey#1) prey protein in Hf7c cells using the Y2H system. (-) indicates no detectable growth of co-transformants on triple drop-out plates after 6–8 days incubation at 30°C while (++) indicates growth after 3–4 days. Results shown are representative of those obtained in 3 independent experiments.

The differential binding of D52-like proteins and MUC1 to MAL2ΔN and MAL (Figure 1b) suggested that D52 and MUC1 bind different MAL2 domains. To map the MUC1 binding domain in MAL2, additional N-terminal MAL2 deletion mutants were constructed, to serially delete the four MAL2 TM domains (Figure 4a). The TM domains were specifically targeted as the MUC1 TM domain was encoded by all prey constructs isolated (Figure 1a). Interactions were detected between MUC1 prey and MAL2ΔN bait, but not MAL2 bait additionally lacking the first TM domain (MAL2ΔNTM1). Another putative MAL2 partner identified through Y2H screening showed detectable interactions with MAL2ΔNTM1, indicating that the lack of MUC1 binding to MAL2ΔNTM1 was not artifactual (S. Fanayan, unpublished results). These analyses therefore indicate that the first MAL2 TM domain (Y^35^-S^56^) is required for MUC1 binding (Figure 4b). Similar deletion mapping experiments involving MUC1 indicated that the minimal MAL2 binding region was contained within the shortest MUC1 prey (prey #7) identified through yeast two-hybrid screening (Figure 1a) (S. Fanayan, unpublished results).

### MAL2 and MUC1 co-localise in the cytoplasm of breast carcinoma cells

**Figure 5:**
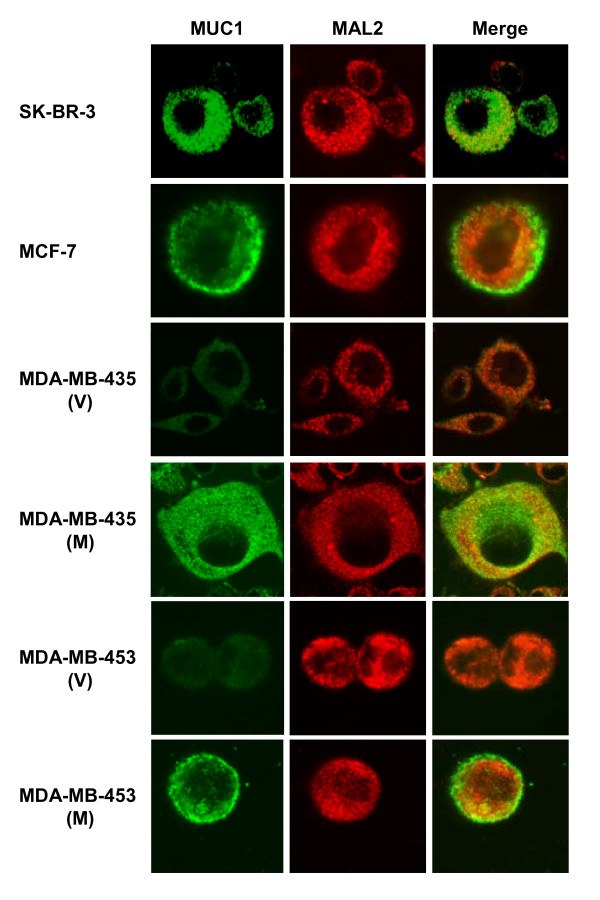
**MAL2 and MUC1 co-localise in the cytoplasm of breast carcinoma cell lines**. Indirect immunofluorescent analyses were carried out using the cell lines indicated to the left of each row, and MUC1 and sheep MAL2 antisera. MUC1 antibody was identified as green color using a FITC-conjugated secondary antibody (left column) and MAL2 antisera was identified as red color using a Cy3-conjugated secondary antibody (middle column). Overlap between MUC1 and MAL2 proteins is shown as yellow in merged images (right column). Results shown represent those obtained from 3–4 independent experiments.

The intracellular localisation of MAL2 in breast cancer cells was determined using indirect immunofluorescence and confocal microscopy, and compared with that of MUC1. As shown in Figure 5, SK-BR-3 and MUC1-overexpressing MDA-MB-435(M) cells showed punctate distributions of MAL2 and MUC1 throughout the cell cytoplasm, with partial co-localisation. In MCF-7 and MDA-MB-453(M) cells, MAL2 was similarly detected throughout the cytoplasm, while MUC1 was concentrated towards the cell periphery, and showed only partial co-localisation with MAL2. These results indicate that MAL2 and MUC1 co-localise within the cytoplasm, regardless of the predominant site of MUC1 localisation. Furthermore, MUC1 overexpression in MDA-MB-435 and MDA-MB-453 cells did not alter MAL2 distribution in these cells (Figure 5).

### MAL2 and MUC1 are present in lipid raft fractions in human breast carcinoma cells

**Figure 6:**
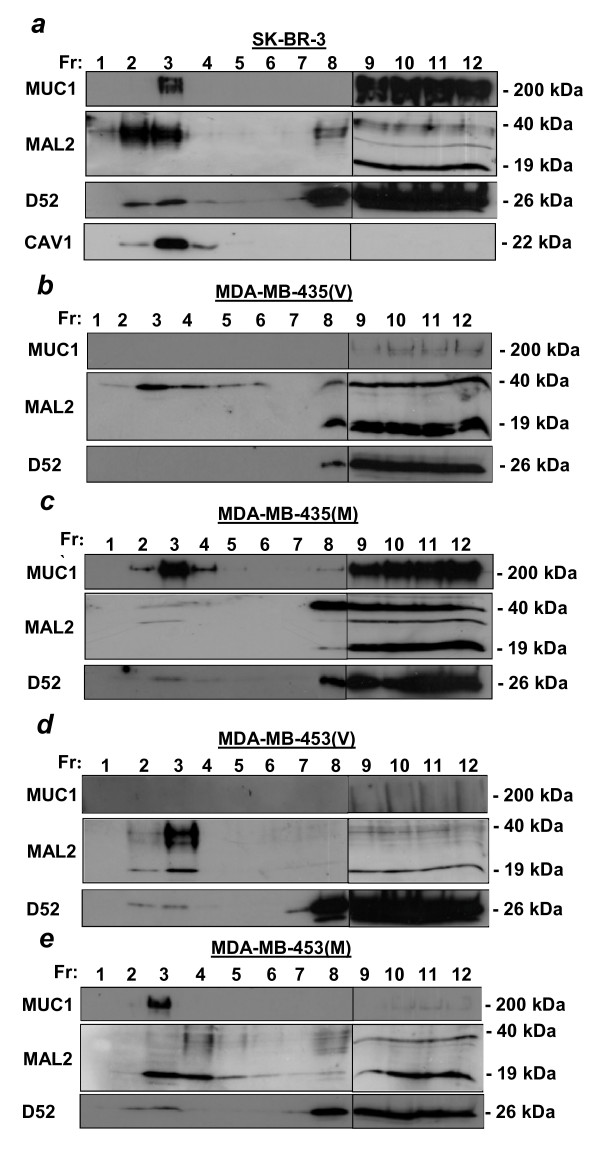
**Distribution of MUC1, MAL2 and D52 in membrane fractions from breast cancer cell lines**. **(a) **SK-BR-3, **(b) **MDA-MB-435(V) **(c) **MDA-MB-435(M) **(d) **MDA-MB-453(V) and **(e) **MDA-MB-453(M) cells were extracted with 1% Triton X-100 at 4°C, and subjected to centrifugation to equilibrium in sucrose density gradients. Twelve 1 ml fractions (Fr, shown above each top panel) were collected from the top of the gradient and 15 μl aliquots from each were subjected to SDS-PAGE and Western blot analysis with antibodies against MUC1, MAL2 and D52, as indicated to the left of each panel. SK-BR-3 fractions **(a) **were also immunoblotted with CAV1 antibody. In all panels, vertical lines between fractions 8 and 9 distinguish samples loaded on different gels. Sizes of detected proteins are indicated at the right. Results shown represent those obtained from 2–3 independent experiments.

MAL2 has consistently been found in lipid raft-containing membrane fractions in hepatoma HepG2 cells [2,14] as well as in human thyroid epithelial cells [15]. We therefore examined the distribution of MAL2 and MUC1 in Triton X-100-soluble and -insoluble fractions from breast carcinoma cell lines. Following centrifugation to equilibrium in sucrose density gradients, the distributions of MAL2 and MUC1 was compared in fractions 2–4, which include Triton X-100-insoluble lipid rafts, and fractions 8–12, which include Triton X-100-soluble cytoplasmic proteins and cell membranes. The distribution of the MAL2 partner D52 was also determined, and compared with that of caveolin-1 (CAV1), a known lipid raft protein [16]. MUC1 was detected in both Triton X-100-soluble and -insoluble fractions when endogenously expressed in SK-BR-3 cells (Figure 6a) and exogenously expressed in MDA-MB-435(M) (Figure 6c) and MDA-MB-453(M) cells (Figure 6e), with MUC1 being predominantly detected in lipid raft fractions in MDA-MB-453(M) cells (Figure 6e). In MDA-MB-435(V) (Figure 6b) and MDA-MB-453(V) cells (Figure 6d), MUC1 was weakly detected in soluble fractions only. MAL2 was detected in both soluble and insoluble fractions in all cell lines examined (Figure 6). The D52 protein was predominantly detected in soluble fractions but weakly detected in insoluble fractions in most cell lines (Figure 6a, b–e). As expected, CAV1 was exclusively detected in lipid raft fractions (Figure 6a and data not shown).

**Figure 7:**
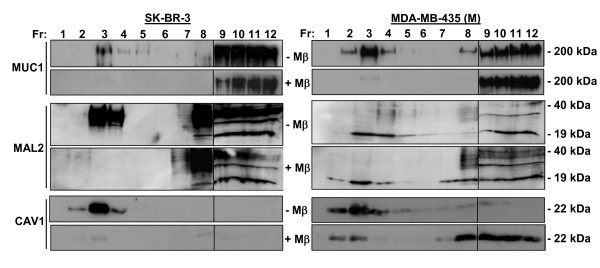
**Effect of MβCD treatment on MUC1 and MAL2 distribution in lipid raft fractions**. SK-BR-3 and MDA-MB-435(M) cells were treated (+Mβ), or not (-Mβ), with 20 mM MβCD, extracted with 1% Triton X-100 at 4°C, and subjected to sucrose gradient centrifugation. Twelve fractions of 1 ml (Fr, as shown above each top panel) were collected and 15 μl aliquots were subjected to SDS-PAGE and Western blot analysis with antibodies to MUC1, MAL2 or CAV1, as indicated at the left, with sizes of detected proteins indicated at the right. In all panels, vertical lines between fractions 8 and 9 distinguish samples loaded on different gels. In fractions 2–4, MUC1, MAL2 and CAV1 were not detected in SK-BR-3 cells, or were significantly reduced in MDA-MB-435 [M] cells following MβCD treatment. Results shown represent those obtained from 3 independent experiments.

**Figure 8:**
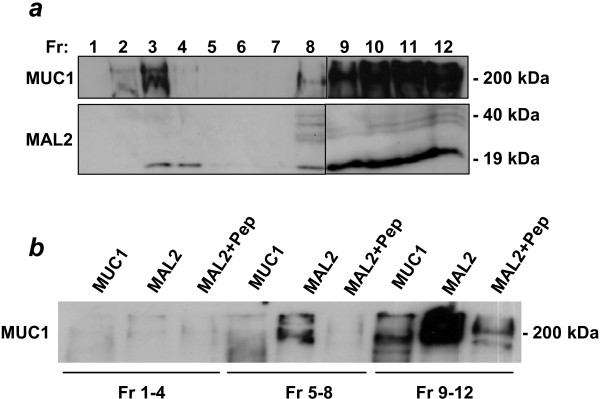
**Co-immunoprecipitation analyses to detect MAL2/MUC1 interactions in Triton X-100-soluble versus-insoluble fractions. MCF-10A/Myc-MAL2 cells were extracted with 1% Triton X-100 at 4°C, and subjected to sucrose gradient centrifugation**. Antisera employed in Western blot analyses are shown at the left, and sizes of detected proteins are shown at the right. **(a) **Fractions (Fr, as shown above the top panel) of 1 ml were collected, and aliquots from each were subjected to SDS-PAGE and Western blot analysis with MUC1 and MAL2 antisera. Vertical lines between fractions 8 and 9 distinguish samples loaded on different gels. **(b) **Pooled fractions (fractions 1–4, which include lipid rafts, fractions 5–8 and fractions 9–12) were immunoprecipitated with MUC1 or MAL2 antisera either alone or with MAL2 peptides (+Pep), as shown at the top of the panel. Immunoprecipitates were separated by SDS-PAGE and subjected to Western blot analysis with MUC1 monoclonal antibody. Results shown represent those obtained from 3 independent experiments.

The effect of cholesterol depletion on MUC1 and MAL2 distributions was examined by treating SK-BR-3 and MDA-MB-435(M) cell lines with MβCD. In SK-BR-3 cells, MβCD treatment rendered MUC1, MAL2 and CAV1 virtually undetectable in fractions 2–4 (Figure 7). The apparent disappearance of CAV1 from SK-BR-3 fractions after MβCD treatment is consistent with CAV1 expression being reported as undetectable in whole SK-BR-3 cell extracts [41,42]. In MDA-MB-435(M) cells, MβCD treatment reduced MUC1, MAL2 and CAV1 detection in the same fractions, which was accompanied by increased detection in Triton X-100-soluble fractions (Figure 7), further supporting co-distribution of MUC1 and MAL2 within lipid rafts. We then carried out (co)-immunoprecipitations of MUC1 and MAL2 from pooled soluble and insoluble membrane fractions from MCF-10A/Myc-MAL2 cells. Both MAL2 and MUC1 were detected in insoluble fractions (Figure 8a), yet MAL2 antisera co-immunoprecipitated MUC1 from pooled soluble fractions only (Figure 8b). The ability of MAL2 antisera to co-immunoprecipitate MUC1 from fractions 5–8 despite low MUC1 concentrations supports an association between MAL2 and MUC1 in Triton X-100 soluble fractions.

### Increased MAL2 expression leads to increased cell surface expression of MUC1

**Figure 9:**
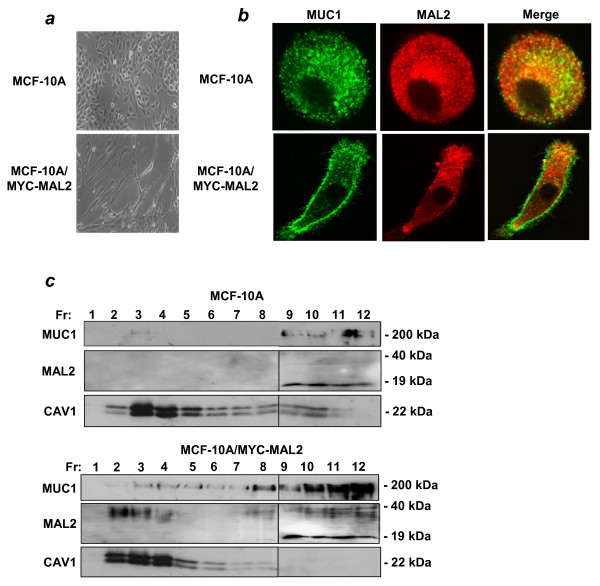
**Effect of ectopic Myc-MAL2 expression on MCF-10A cell morphology and intracellular localisation of MUC1**. **(a) **MCF-10A and MCF-10A/Myc-MAL2 cells were grown in culture and their morphologies compared by light microscopy (X100 magnification). **(b) **MCF-10A and MCF-10A/Myc-MAL2 cells were co-stained for MUC1 (left panel) and MAL2 (middle panel). Overlap between the MUC1 and MAL2 proteins are shown in merged images (right panel). Images shown are single horizontal x-y sections. **(c) **MCF-10A and MCF-10A/Myc-MAL2 cells were extracted with 1% Triton X-100 at 4°C, and centrifuged to equilibrium in sucrose density gradients. Twelve fractions of 1 ml were collected (Fr, as shown above each top panel), and aliquots were subjected to SDS-PAGE and Western blot analysis with MUC1, MAL2 or CAV1 antisera, as indicated at the left, with sizes of detected proteins indicated at the right. Vertical lines between fractions 8 and 9 distinguish samples loaded on different gels. Results shown represent those obtained from 3 independent experiments.

Interactions between MUC1 and MAL2, together with previous data showing MAL2 involvement in basolateral-to-apical transport [2] suggested a role for MAL2 in localising MUC1 to the cell surface. The effect of ectopic Myc-MAL2 expression on MUC1 subcellular localisation was therefore compared in parental and MCF-10A/Myc-MAL2 cells. As shown in Figure 9a, MCF-10A/Myc-MAL2 cells showed a dramatically elongated morphology relative to the parental cell line, which did not reflect a change in differentiation status, as assessed using neuronal and epithelial-mesenchymal transition markers (data not shown). Increased detection of glycosylated MAL2 in both raft and non-raft fractions from MCF-10A/Myc-MAL2 cells was also accompanied by increased MUC1 detection across all membrane fractions (Figure 9c). Comparing the sub-cellular localisations of these proteins using confocal microscopy revealed an accumulation of MUC1 at the periphery of MCF-10A/Myc-MAL2 cells compared with parental cells, which incompletely co-localised with MAL2 (Figure 9b). Taken together, these data show that ectopic Myc-MAL2 expression in MCF-10A/Myc-MAL2 cells expands the plasma membrane domain, and increases MUC1 detection at the cell periphery.

## Discussion

MAL2 was first identified through its expression in breast carcinoma and interactions with D52-like proteins within the Y2H system [1]. We therefore undertook a Y2H screen to identify MAL2 binding partners expressed in human breast cancer tissue, which identified a number of novel putative binding proteins, including MUC1. MUC1 represented a candidate partner of particular interest, given the fact that it is overexpressed in cancer types where MAL2 overexpression has also been reported, and as MUC1 overexpression contributes to cancer progression through a number of mechanisms [43,44]. Interactions between human MUC1 and MAL2 proteins are also broadly consistent with the previously reported interactions between the yeast signalling mucin Msb2 and the tetraspanin protein Sho1 [45]. Subsequent results collectively identify MAL2 as a novel cytoplasmic MUC1 partner, and a possible regulator of MUC1 expression and/or subcellular distribution. It is striking to note that MAL2, a chromosome 8q24 amplification target, has now been shown to bind MUC1 and D52, both of which are amplified and/or overexpressed in breast and other cancers [21,22,34,35,46] strongly suggesting that these proteins have co-operating functions in cancer cells.

While a recent study by Kinlough *et al*. [47] indicated that palmitoylation is the dominant feature modulating MUC1 recycling to the plasma membrane, the mechanisms by which MUC1 is targeted and maintained at the plasma membrane are not fully understood. Since ectopic Myc-MAL2 expression in MCF-10A cells was associated with increased MUC1 detection at the cell periphery in the present study, MAL2 may play a direct or indirect role in MUC1 targeting. Interestingly, MAL2 incompletely co-localised with peripheral MUC1 in MCF-10A/Myc-MAL2 cells, which supports previous findings that altering MAL2 expression can alter cargo accumulation distant to the predominant site of MAL2 expression [2,14]. However, ectopic Myc-MAL2 expression also produced an apparent expansion of cell surface domains in MCF-10A cells, which may also contribute to increased MUC1 detection at the cell periphery. Similar observations have been made in a previous study where MAL overexpression altered the morphology of MDCK cells, by seemingly expanding apical cell surface domains through increased apical delivery [6]. Finally, we also noted that ectopic MAL2 expression produced an increase in MUC1 detection across Triton X-100-soluble and -insoluble protein fractions in MCF-10A/Myc-MAL2 cells, indicating that Myc-MAL2 may positively regulate MUC1 expression. This may also lead to increased peripheral MUC1 detection. While it remains to be determined whether MAL2 can alter MUC1 distribution in other cell types, the broad expression of MAL2 within epithelia [13] is consistent with MAL2 contributing to MUC1 targetting under physiological conditions. It will also be of interest to examine whether MAL2 can similarly regulate MUC1 secretion.

If MAL2 similarly regulates MUC1 expression and/or distribution in cancer cells, increased MAL2 levels could alter cancer cell biology in several ways. Increased MUC1 localisation at the cell surface could reduce intercellular adhesion and promote invasiveness [44], and increased MAL2 expression would thus be expected to have adverse significance in cancer cells. Accordingly, *MAL2 *has been included within a gene signature of poor prognosis in breast cancer [48], and *MAL2 *overexpression was associated with resistance to doxorubicin therapy in breast cancer patients [49]. MAL2 overexpression might also reduce cytoplasmic MUC1 accumulation, which has been indicated to be an adverse finding in breast and ovarian carcinomas [50-52], tumor types which also overexpress *MAL2 *[20,24,25]. Interestingly, suppression of *MUC1 *expression in a pancreatic cancer cell line reduced these cells' metastatic potential, and was accompanied by reduced *MAL2 *levels [53]. Further direct analyses are therefore required to determine whether MAL2 and MUC1 levels are positively correlated in cancer types commonly expressing these proteins, and whether MAL2 expression is significantly associated with cell surface expression of MUC1 in cancer cells.

The present study also provides the first report that MUC1 localises within lipid raft fractions in breast carcinoma cells, and raises the possibility that some of MUC1's signalling functions [43,44] may occur within lipid rafts. Previous studies reported that MUC1 in T-lymphocyte cell lines was insoluble in cold Triton X-100 and associated with low density membrane fractions [54,55], yet Kinlough *et al*. [47] showed MUC1 from MDCK cells was fully soluble in Triton X-100. The reported differences in MUC1 solubility may be due to cell specific differences in membrane composition and their selectivity for detergents, as reported by Schuck *et al*. [56], or alternatively MUC1 may not reside in lipid rafts in all cell types. We demonstrated associations between MUC1 and MAL2 in Triton X-100 soluble fractions, in agreement with MUC1 and MAL2 being predominantly detected in non-raft fractions in all cell lines analysed, which was also noted for the MAL2 partner D52. While we were unable to determine whether MAL2 and MUC1 associate within lipid rafts, our results indicate that MAL2 associates with MUC1 and potentially other proteins outside lipid raft membrane microdomains, as indicated for other tetraspanins [57], and highlights the fact that MAL2 may have independent functions within and outside lipid rafts.

Our analysis of MAL2 partners has also indicated that MAL2 may represent a multifunctional transmembrane adaptor protein, capable of binding more than one partner simultaneously. We noted that both MAL2 and D52 co-immunoprecipitated with MUC1 in the present study, despite the fact that direct interactions between MUC1 and D52 were not detected in the Y2H system. Independent interaction domains for MAL2/MUC1 and MAL2/D52 binding were also indicated by the MAL2 N-terminal domain being required to bind D52-like proteins, yet the first TM domain was required for binding MUC1. Correspondingly, D52-like proteins did not bind MAL, whose N-terminal sequence is poorly conserved with respect to that of MAL2 [1], yet MUC1 bound both MAL and MAL2 in the Y2H system. These results therefore predict shared and isoform-specific functions for MAL-like proteins, through shared and discrete binding partners.

## Conclusion

This study has identified MAL2 as a novel cytoplasmic MUC1 partner and a potential regulator of MUC1 subcellular distribution. MAL2/MUC1 interactions were detected in Triton X-100-soluble membrane fractions, indicating that MAL2 has specific functions within non-raft membrane compartments in breast cancer cells. Since *MAL2 *is known to be amplified and overexpressed in breast and other cancers, it is striking that MAL2 has now been shown to bind two proteins, MUC1 and D52, which are also amplified and/or overexpressed. Based on these results, and a previous study highlighting the co-amplification of *MAL2 *and *D52 *genes in breast cancer [21], it will be interesting to examine whether *MAL2 *and *MUC1 *are commonly amplified and overexpressed in breast cancer, and whether MAL2 expression is significantly associated with cell surface expression of MUC1.

## Abbreviations

TM: transmembrane; EGF: epidermal growth factor; CKLFSF8: chemokine-like factor superfamily8; Y2H: yeast two-hybrid; cfu: colony forming unit; MAL: Mal T-cell differentiation protein; MUC1: mucin 1; CAV1: caveolin-1; PCR: polymerase chain reaction; BSA: bovine serum albumin; MβCD: methyl β-cyclodextrin.

## Competing interests

The authors declare that they have no competing interests.

## Authors' contributions

SF contributed to the Y2H screening and analyses, carried out all other experiments, and drafted the manuscript. MS assisted with Y2H screening, and MS and APA contributed to subsequent Y2H analyses. MAM and MAA provided essential reagents for the analysis of MUC1 and MAL2, respectively, and participated in the design of the study. JAB conceived the study, conducted the Y2H screen, participated in study design and co-ordination, and helped to draft the manuscript. All authors read and approved the final manuscript.
